# Safety and Immunogenicity of Vaccines in Children with Kaposiform Hemangioendothelioma Receiving Sirolimus: A Prospective Study

**DOI:** 10.3390/vaccines13090903

**Published:** 2025-08-26

**Authors:** Junhong Yuan, Zhenxiang Yuan, Yingjing Ding, Zuopeng Wang, Wei Yao, Jingjing Li, Mei Zeng, Kai Li

**Affiliations:** 1 Department of Pediatric Surgery, Children’s Hospital of Fudan University, Shanghai 201100, China; 2 Department of Infectious Diseases, Children’s Hospital of Fudan University, Shanghai 201100, China

**Keywords:** kaposiform hemangioendothelioma, sirolimus, vaccination, immunogenicity, safety, pediatric vascular tumor

## Abstract

Background: Sirolimus is an effective treatment for kaposiform hemangioendothelioma (KHE), a rare vascular tumor in children. However, its immunosuppressive properties raise concerns regarding the safety and efficacy of vaccinations during treatment. This study aims to evaluate the safety and immunogenicity of inactivated and live-attenuated vaccines administered to pediatric KHE patients undergoing sirolimus therapy. Methods: We conducted a prospective study involving 56 KHE children receiving sirolimus who were vaccinated during treatment. Data on vaccine-related adverse events were collected to assess safety. Immunogenicity was evaluated by measuring seroconversion or protective antibody titers against vaccines, including Hepatitis B, DTaP, and MMR. Results: Among 56 catch-up vaccinated children, no serious adverse events related to vaccination were observed. Mild local or systemic reactions occurred in a minority of patients. Serological analysis demonstrated that children with kaposiform hemangioendothelioma (KHE) receiving sirolimus therapy were able to generate and sustain robust protective antibody responses following vaccination. High seroconversion rates and antibody titers were observed for both inactivated vaccines (e.g., hepatitis B and DTaP) and live-attenuated vaccines (e.g., MMR). Protective antibody levels were maintained both within 3 months and beyond 6 months post-vaccination, indicating durable immunogenicity under sirolimus treatment. Conclusions: Vaccination during sirolimus therapy appears to be safe and immunogenic in children with KHE. These findings support the administration of both inactivated and live-attenuated vaccines under appropriate clinical monitoring in this rare patient population.

## 1. Introduction

Sirolimus (rapamycin), an mTORC1 inhibitor targeting the PI3K-AKT-mTOR signaling pathway, was approved by the U.S. Food and Drug Administration in 1999 as an immunosuppressive agent. Since 2010, accumulating clinical evidence has demonstrated its superior therapeutic efficacy in the management of kaposiform hemangioendothelioma (KHE), a rare pediatric vascular tumor. Sirolimus has been shown to significantly improve clinical symptoms, elevate platelet counts, and restore coagulation function in affected patients [1]. Notably, it achieves high remission rates in cases refractory to conventional therapies such as corticosteroids and vincristine, or in patients who experience relapse following dose tapering. These findings support the growing consensus that sirolimus is emerging as the first-line treatment for KHE [2]. Individuals receiving immunosuppressive agents often experience a certain degree of immune dysfunction compared to healthy populations, making them more susceptible to infectious diseases. For example, pediatric organ transplant recipients require long-term—even lifelong—use of immunosuppressants, with infectious complications being the leading cause of morbidity and mortality among all transplant recipients [3]. In China, missed vaccines and delayed vaccinations are common among children with special health conditions due to the use of immunosuppressive drugs [4].

Given the immunosuppressive properties of sirolimus, vaccination is also generally not recommended during sirolimus treatment in pediatric patients. But as research on sirolimus continues to deepen, its impact on T cells, particularly memory CD8 + cells, has shown bidirectional effects, which are dependent on both the dosage and duration of use [5]. Studies have reported that the use of low-dose sirolimus can enhance the immune response to vaccination, leading to more sustained immune protection. This effect is largely dependent on the dosage, as high-dose sirolimus may suppress T cell proliferation [6].

However, both domestic and international studies evaluating the safety and immunogenicity of vaccines administered during sirolimus therapy in children remain limited. The absence of vaccine-induced immune protection may increase the risk of vaccine-preventable infections (VPIs) in children with kaposiform hemangioendothelioma (KHE) receiving sirolimus [7]. Therefore, determining the optimal vaccine formulation and timing of administration is crucial for reducing the risk of VPIs and improving infection prevention strategies in this vulnerable population.

Building upon previous exploratory work conducted in a small cohort, this study aims to systematically assess the efficacy and safety of pediatric vaccinations in children with kaposiform hemangioendothelioma (KHE) receiving sirolimus treatment. The objective is to generate clinical evidence to inform vaccination strategies for this specific patient population. With support from our center’s Vaccination Evaluation Clinic, a more comprehensive investigation has been undertaken to expand upon earlier findings and provide practical guidance for immunization in the context of sirolimus therapy.

## 2. Methods

### 2.1. General Information

From January 2017 to December 2024, children diagnosed with kaposiform hemangioendothelioma (KHE) were retrospectively screened from both inpatient and outpatient records at our hospital. The inclusion criteria were: (1) diagnosis of KHE according to the 2013 consensus guidelines on the diagnosis and treatment of KHE and Kasabach–Merritt phenomenon (KMP) [8]; (2) regular treatment with sirolimus monotherapy; and (3) clinically stable disease, defined as the absence of tumor enlargement, hemorrhagic tendency, or occurrence of KMP.

Exclusion criteria included: (1) diagnosis of other vascular tumors such as infantile hemangiomas or tufted angiomas; (2) receipt of surgical excision, laser therapy, topical agents, or other systemic therapies (e.g., etoposide, corticosteroids) in addition to sirolimus; (3) disease progression or coexistence of serious comorbid conditions; (4) inability to provide reliable clinical and vaccination history; (5) disease deterioration during follow-up (e.g., tumor enlargement, bleeding tendency); and (6) occurrence of serious adverse events following vaccination (e.g., anaphylactic shock or sustained organ dysfunction).

A total of 56 children met the inclusion criteria and were enrolled in the study. Ethical approval was obtained from the Ethics Committee of the Children’s Hospital of Fudan University. The study was conducted in accordance with the Declaration of Helsinki, and written informed consent was obtained from the parents or legal guardians of all participants.

### 2.2. Subject Grouping

The enrolled children were categorized into a routine vaccination group and a catch-up vaccination group based on the timing of immunization relative to the recommended age-specific vaccination schedule outlined in the Chinese National Immunisation Programme Vaccine Immunisation Procedure Table for Children (2021 edition), as well as the timing of sirolimus initiation.

The routine vaccination group included children who had completed the corresponding vaccine doses within one month of the recommended age as stipulated in the 2021 national immunization schedule, prior to the initiation of sirolimus therapy.

The catch-up vaccination group comprised children who did not receive the corresponding vaccine doses within one month of the recommended schedule and instead received catch-up immunization during sirolimus treatment to complete the vaccination series.

### 2.3. Vaccination and Catch-Up

The vaccination status of children with kaposiform hemangioendothelioma (KHE) was evaluated based on the age-specific immunization schedule outlined in the National Immunisation Programme Vaccine Schedule for Children (2021 edition), and catch-up vaccination was administered accordingly.

### 2.4. Observation Indicators

Clinical and laboratory data from 56 children were collected before and after vaccination within 3 months and beyond 6 months. Clinical and laboratory features include routine hematological parameters, coagulation profiles, and immunological indicators. Specifically, these included complete blood count, key coagulation markers (e.g., APTT, PT, fibrinogen, D-dimer), and serologic levels of hepatitis B surface antibody (HBsAb) and IgG antibodies against common childhood infections (e.g., pertussis, diphtheria, tetanus, measles, rubella, and mumps). Prior to vaccination, assessments also included CD lymphocyte subsets, immunoglobulin levels, and complement concentrations. Adverse reactions were recorded for each vaccine dose, including mild events (e.g., fever, injection-site pain, erythema, and swelling) and serious events (e.g., severe anaphylaxis, shock, lesion enlargement, and lesion-site pain).

### 2.5. Statistical Analysis

Quantitative data are presented as mean ± standard deviation (SD). For comparisons of continuous variables between two independent groups with homogeneity of variance, independent samples *t*-tests were applied. Categorical variables were reported as frequencies or percentages, and group comparisons were conducted using the chi-square test or Fisher’s exact test, as appropriate. Paired *t*-tests were used to assess changes in antibody levels and laboratory parameters before and after vaccination. All statistical analyses and graphical outputs were performed using SPSS Statistics version 17.0 (IBM Corp., Armonk, NY, USA) and GraphPad Prism 10.1.2 (GraphPad Software, San Diego, CA, USA).

## 3. Results

### 3.1. Demographic and Clinical Characteristics

**Figure 1:**
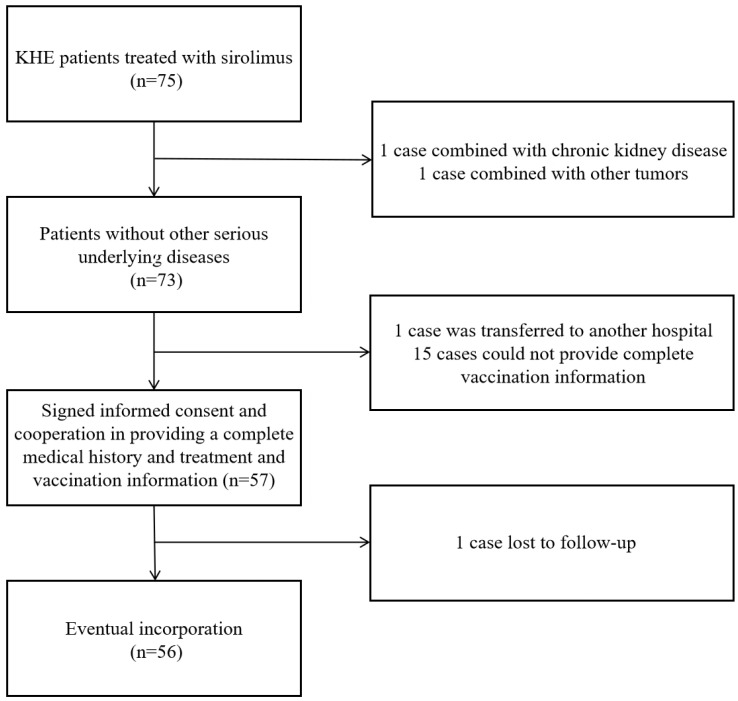
Inclusion and exclusion criteria for enrollment.

From January 2017 to December 2024, a total of 56 children diagnosed with kaposiform hemangioendothelioma (KHE) and treated with sirolimus were enrolled in this study through outpatient and inpatient screening at the Children’s Hospital of Fudan University, based on the predefined inclusion criteria (Figure 1). Among them, 32 were male and 24 were female. All enrolled patients were treated according to the consensus statement on the diagnosis, treatment, and prognosis of kaposiform hemangioendothelioma (KHE), which was co-authored by our team [9]. Sirolimus therapy was initiated at a dose of 0.8 mg/m^2^ per day, followed by a 1-week administration period. Thereafter, sirolimus dosage was adjusted monthly based on trough blood levels, aiming to maintain a target serum trough concentration between 5 and 15 ng/mL. Baseline demographic and clinical characteristics, as well as pre-catch-up vaccination status, are summarized in Table 1 and Supplementary Table S1. Based on our continuous monitoring and evaluation of sirolimus blood concentration levels in all catch-up vaccination patients prior to receiving their first vaccination, all patients had stable sirolimus blood concentration levels within the range of 3.5–10.0 ng/mL at the start of the vaccination process. Some patients exhibited relatively low sirolimus blood concentration levels because they were in a tapering maintenance phase of treatment. Compared with the reported delayed or missed vaccination rates among healthy children in China (ranging from 0.047% to 5.29%) [10], the children in this study exhibited substantially higher rates of missed vaccinations.

### 3.2. Safety Evaluation of Vaccination

During sirolimus treatment, the mean percentages of T and B lymphocytes, as well as immunoglobulin and complement levels in children with KHE, remained within the normal reference ranges, as determined by CD lymphocyte subset analysis and immunological profiling. These values were comparable to those observed in age-matched healthy children (Supplementary Table S2).

Following vaccination, a subset of patients experienced mild adverse reactions, including local pain, erythema, and swelling at the injection site. Importantly, no serious adverse events such as severe allergic reactions, anaphylaxis, lesion enlargement, or lesion-associated pain were reported in any participant (Table 2).

Whether the lesion has enlarged is determined by comparing the MRI scan taken before the vaccine was administered with one taken within one year afterwards. Pain at the site of the lesion is determined by the child’s main complaint, as assessed by the doctor.

**Figure 2:**
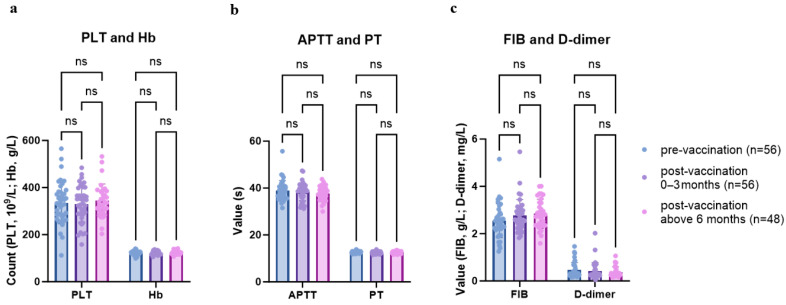
Changes in complete blood count (CBC) and coagulation function before and after vaccination. ns, not significant.

In addition, post-vaccination assessments revealed no significant changes in platelet count, hemoglobin concentration, or coagulation parameters compared with pre-vaccination values (*p* > 0.05) (Figure 2, Supplementary Table S3). All hematological and coagulation indices remained within normal limits. No cases of Kasabach–Merritt phenomenon were observed during the follow-up period.

### 3.3. Effectiveness Evaluation of Vaccination

Among the 56 children included in the study, 15 had completed age-appropriate hepatitis B vaccination prior to sirolimus therapy and were classified as the normal vaccination group. The remaining 41 children, who had not received hepatitis B vaccination within the recommended age window, underwent catch-up vaccination after initiation of sirolimus treatment. Among them, three failed to complete the third dose of the hepatitis B vaccine. No statistically significant differences were observed in age or sex distribution between the normal and catch-up groups (*p* = 0.093).

**Figure 3:**
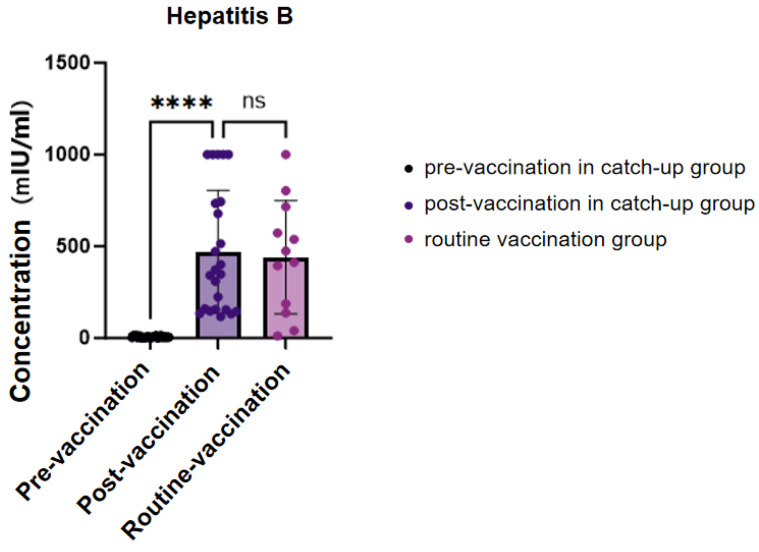
HBs-Ab concentration level before and after Hepatitis B vaccination. ns, not significant, ****, *p* < 0.0001.

Following catch-up vaccination, the hepatitis B surface antibody (HBs-Ab) concentrations in the catch-up group increased significantly and reached levels comparable to those observed in the routine vaccination group. Notably, the post-vaccination HBs-Ab concentrations in the catch-up group were significantly higher than their pre-vaccination levels in the catch-up group (*p* < 0.01; 95% CI [322.33–605.25]) (Figure 3, Supplementary Table S4).

For diphtheria-tetanus-pertussis (DTP) vaccination, 16 children had completed the age-appropriate immunization schedule prior to sirolimus administration and were classified into the normal vaccination group. A total of 37 children received DTaP catch-up vaccination after initiation of sirolimus therapy due to delayed immunization, while 3 children had not received the DTaP vaccine during the study period. No statistically significant differences in age or sex were observed between the normal and catch-up groups (*p* = 0.536).

**Figure 4:**
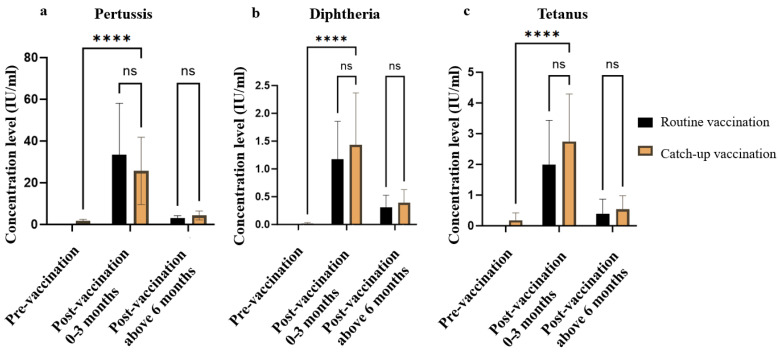
Levels of antibody concentration before and after DTaP vaccination. ns, not significant, ****, *p* < 0.0001.

Within 0–3 months following catch-up vaccination, antibody concentrations in the DTaP catch-up group showed a significant increase compared to pre-vaccination levels (*p* < 0.01) and reached comparable levels to those of the normal vaccination group. However, at more than 6 months post-vaccination, a marked decline in antibody titers was observed for tetanus, with only two children maintaining immunoprotective levels. In contrast, the majority of children in the catch-up group retained immunoprotective levels of antibodies against pertussis and diphtheria. A similar pattern of antibody waning was also observed in the normal vaccination group. (Figure 4, Table 3 and Supplementary Table S5)

Moreover, the impact of age and sirolimus duration on antibody titers are investigated after catch-up vaccination. Multivariate linear regression (Supplementary Table S6) revealed that each additional year of sirolimus exposure statistically significantly reduced pertussis and tetanus IgG titers. Survival analysis (Supplementary Table S7) further showed that sirolimus accelerated the loss of protective immunity, particularly for tetanus.

Given that the recommended ages for the first and second doses of the measles–mumps–rubella (MMR) vaccine are 8 months and 18 months, respectively, and that the mean age at disease onset in children with KHE in this cohort was 5.2 ± 11.0 months, the MMR vaccine deficiency rate at the time of diagnosis was 94.6%. Among the 56 children included, 53 who had not completed MMR vaccination underwent catch-up immunization, and 47 of these received the vaccine while on sirolimus treatment. However, the sample sizes between the two-dose (n = 30) and one-dose (n = 6) MMR vaccine groups was imbalance because the majority of children in this study had already received two doses of the MMR vaccine at the time of assessment, while only six children had received a single dose for various reasons. Given this imbalance, the statistical comparisons between these groups should be interpreted with caution, as the small sample size in the one-dose group may affect the robustness of the analysis.

**Figure 5:**
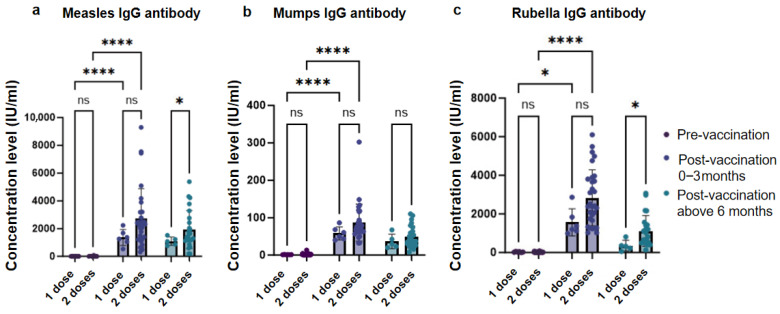
Levels of antibody concentration before and after MMR vaccination. ns, not significant, *, *p* < 0.05, ****, *p* < 0.0001.

Within 0–3 months after MMR catch-up vaccination, serum IgG antibody levels against measles, mumps, and rubella significantly increased compared to pre-vaccination levels (*p* < 0.01). At 6 months post-vaccination, although a decline in antibody titers was observed, both the proportion of children maintaining seroprotective levels and the absolute antibody concentrations remained relatively high (Figure 5, Supplementary Table S8).

## 4. Discussion

As an mTOR inhibitor, sirolimus effectively inhibits protein synthesis in the PI3K–AKT–mTOR signaling cascade, thereby suppressing cellular proliferation and angiogenesis [11]. It has increasingly been adopted as a first-line therapy for vascular tumors such as kaposiform hemangioendothelioma (KHE) [12]. However, due to its immunosuppressive properties, delayed or missed vaccinations are commonly observed among children with special health conditions requiring immunosuppressive therapy in China. In the present study, a high prevalence of missed vaccinations was observed among children with KHE receiving sirolimus. Notably, the omission rates for several live attenuated vaccines—including the measles–mumps–rubella (MMR) vaccine, meningococcal vaccine, and live attenuated Japanese encephalitis vaccine—ranged from 80% to 97.5%, which may substantially increase the risk of vaccine-preventable infections (VPIs) in this population.

### 4.1. Safety Evaluation of Vaccination

The immunosuppressive treatment regimen and dosage can exert a significant impact on the baseline immune function of pediatric patients. It is generally accepted that for children with hematological malignancies, at least six months after the completion of chemotherapy are required for the immune system to recover sufficiently to elicit an adequate immune response to vaccination [13].

According to the 2013 guidelines of the Infectious Diseases Society of America (IDSA), administration of live attenuated vaccines is contraindicated in hematopoietic stem cell transplant recipients undergoing immunosuppressive therapy. Similarly, for patients with chronic inflammatory diseases, live attenuated vaccines such as measles-mumps-rubella (MMR) and varicella are not recommended during the maintenance phase of immunosuppressive treatment. However, for patients receiving long-term low-dose immunosuppressive therapy who have no prior immunity to varicella, vaccination may be considered [14]. These recommendations highlight the potential risk associated with live attenuated vaccines in immunocompromised pediatric patients, who may not only be susceptible to vaccine-related adverse effects but may also serve as potential sources of transmission of vaccine-derived pathogens within the community.

In this study, assessment of CD lymphocyte subsets, immunoglobulin levels, and complement concentrations prior to catch-up vaccination in 56 children with KHE revealed that the majority of patients receiving low-dose sirolimus maintained normal baseline immune function. These findings suggest that during sirolimus treatment, the T and B cell function, as well as immunoglobulin and complement levels in children with KHE, are comparable to those in healthy children. This serves as an important rationale for proposing that children with KHE undergoing sirolimus therapy may be eligible for routine vaccination.

Our results further demonstrated that both inactivated and live attenuated vaccines were well tolerated in this cohort. Most adverse reactions were mild and transient, with no serious adverse events such as anaphylaxis, shock, lesion enlargement, or lesion-site pain observed. Importantly, no vaccine-related infections occurred following immunization, indicating that vaccination during sirolimus treatment is not associated with increased infectious risk in this population.

Follow-up assessments of platelet count, hemoglobin concentration, activated partial thromboplastin time (APTT), prothrombin time (PT), fibrinogen (FIB) level, and D-dimer concentration before and after vaccination in children with KHE undergoing sirolimus treatment demonstrated that all values remained within normal reference ranges, with no significant changes observed post-vaccination (all *p*-values > 0.05). These findings indicate that vaccination does not exert a significant impact on platelet levels, hemoglobin concentration, or coagulation function in this population. Moreover, vaccination did not induce the occurrence of Kasabach–Merritt phenomenon in any patient.

### 4.2. Effectiveness Evaluation of Vaccination

The findings of this study indicate that children with KHE receiving sirolimus treatment were capable of mounting effective and protective immune responses following vaccination with both inactivated and live attenuated vaccines. Within three months and beyond six months after administration of inactivated vaccines such as hepatitis B and DTaP, the concentrations of HBsAb, pertussis IgG, diphtheria IgG, and tetanus IgG antibodies remained at high and protective levels. Similarly, live attenuated vaccines, including the MMR vaccine, also elicited robust immune responses, with seroconversion rates of measles IgG, rubella IgG, and mumps IgG antibodies approaching 100% within three months post-vaccination. Protective antibody titers were largely maintained beyond six months after vaccination.

Notably, the magnitude and persistence of antibody responses observed in this cohort were superior to those reported in previous studies involving immunocompromised children undergoing chemotherapy, hematopoietic stem cell transplantation, or treatment with other immunosuppressants [15,16]. This discrepancy may be attributed to the relatively low dosage of sirolimus administered to KHE patients, as well as the distinct disease characteristics compared to those of other immunosuppressed populations. These factors may contribute to the more favorable immunogenicity observed in KHE children receiving sirolimus therapy.

Although antibody levels in KHE patients receiving sirolimus therapy tend to decline over time, most vaccine-specific antibodies—including HBs-Ab, pertussis IgG, diphtheria IgG, measles IgG, rubella IgG, and mumps IgG—remained above protective thresholds for effective immunity at least six months after vaccination. Notably, only tetanus IgG exhibited a marked seroreversion, while no significant loss of serological protection was observed for the other antibodies.

Notably, the survival analysis revealed hazard ratios (HR) of 1.28 for pertussis IgG, 1.21 for diphtheria IgG, and a notable 1.40 for tetanus IgG, indicating an accelerated loss of protective immunity, particularly for tetanus. These findings suggest the need for closer monitoring and potentially more frequent booster doses to maintain adequate protection in patients on long-term sirolimus therapy.

Emerging evidence suggests that different mTOR inhibitors may have distinct immunomodulatory effects relevant to vaccine responsiveness. In kidney transplant recipients, sirolimus-based immunosuppression was associated with significantly enhanced SARS-CoV-2–specific T-cell responses and higher frequencies of functional memory T cells, particularly within CD4^+^ and CD8^+^ compartments, compared to standard calcineurin inhibitor regimens [17]. Similarly, temsirolimus has been shown in murine tumor vaccine models to promote CD8^+^ T cell memory and increase IFN-γ production, thereby enhancing long-term immune protection without impairing recall capacity [18]. Moreover, rapamycin, a prototypical mTOR inhibitor, was found to boost long-lived T-cell memory and cytokine recall responses when used as an adjuvant to tuberculosis subunit vaccines in mice [19]. These findings underscore the potential of mTOR inhibition to modulate adaptive immune memory in favor of improved vaccine efficacy and provide a useful contextual framework for interpreting our findings, supporting the broader investigation of mTOR inhibitors in vaccine optimization strategies.

According to the 2017 World Health Organization position paper on tetanus vaccination [20], lifelong protection against tetanus typically requires six doses of tetanus toxoid–containing vaccines—three primary doses followed by three booster doses. For individuals who receive only the three-dose primary series and fail to achieve long-term protective antibody levels, a booster dose of a combined tetanus-diphtheria-pertussis (Tdap) vaccine is recommended. In our study, most patients had completed only the three-dose primary series, which may explain the marked decline in tetanus IgG antibodies observed in both the routinely vaccinated and catch-up groups more than six months after DTaP vaccination. We believe that for children receiving immunosuppressive therapy, a personalized tetanus booster strategy should be developed. As the immune responses in these children may be weakened, the standard vaccination schedule may not provide sufficient protection. In this study, some patients, despite having a vaccination history, still exhibited suboptimal antibody levels. This finding suggests that additional booster doses should be considered for such populations. This viewpoint aligns with the WHO’s recommendation to tailor vaccination plans based on individual risk and immune status.

A previously published multicenter study reported that 93% of children with inflammatory bowel disease, regardless of immunosuppressive treatment status, developed protective immunity following Tdap booster immunization [21]. These findings suggest that seronegative children after primary DTaP vaccination may regain protective tetanus immunity through administration of a Tdap booster dose.

This study has some limitations in this study. One is the imbalanced sample sizes between the normal vaccination group and the supplemental vaccination group for both Hepatitis B (n = 12 vs. n = 24) and DTaP (n = 10 vs. n = 24). This imbalance was a result of the grouping being based on the actual vaccination status of the enrolled patients, leading to unequal sample sizes. Our post hoc statistical power analysis confirmed that the comparison between these two groups had limited statistical power (0.32–0.57), which may impact the reliability of the observed results. We recognize this limitation and emphasize the need for a larger study to validate our findings and further evaluate the immunogenicity and safety of vaccination in this patient population. Moreover, as this study did not measure sirolimus levels during each vaccination in patients, further research and optimization of the study design are needed to clarify the correlation between sirolimus levels and antibody titers.

Currently, there is limited research on the safety and efficacy of vaccinations in children with kaposiform hemangioendothelioma (KHE) undergoing sirolimus therapy, and the existing literature tends to adopt a conservative stance regarding immunization in this population. However, the high rate of missed vaccinations and the consequent increased risk of vaccine-preventable infections in these patients highlight the urgent need to investigate the immunogenicity and safety of routine vaccines in this group. Our findings suggest that both inactivated and live attenuated vaccines can elicit effective and sustained immune responses in KHE patients receiving sirolimus treatment. Nonetheless, due to the limited duration of follow-up in this study, we were unable to fully assess the long-term persistence of immune memory. Future studies with extended follow-up periods are warranted to better evaluate the durability of vaccine-induced immunity during sirolimus therapy.

## 5. Conclusions

This study provides important insights into the safety and immunogenicity of vaccinations in children with kaposiform hemangioendothelioma (KHE) receiving sirolimus therapy. Our findings demonstrate that both inactivated and live-attenuated vaccines can elicit effective and sustained immune responses in this pediatric population under immunosuppressive treatment. Importantly, no serious adverse events were observed following vaccination, indicating that immunization during sirolimus therapy is safe.

This study further highlights that despite the immunosuppressive effects of sirolimus, children with KHE can still maintain a robust immune response to vaccines, with seroprotective antibody levels sustained over a significant period. However, some vaccine responses, particularly to tetanus, showed signs of waning over time, underlining the importance of monitoring and adjusting vaccination schedules for children on immunosuppressive therapies.

Our results support the continuation of routine vaccinations during sirolimus treatment in children with KHE, with careful clinical monitoring. Further research with longer follow-up periods and larger sample sizes is necessary to fully assess the long-term durability of immune responses and optimize vaccination strategies for this vulnerable population. Additionally, personalized vaccination strategies, particularly regarding tetanus boosters, should be considered for children receiving immunosuppressive therapy.

## Figures and Tables

**Table 1:** Basic characteristics of children with KHE prior to vaccine catch-up.

Features	Numerical Value
Sex (number, n)	
male	32
female	24
Age (mean ± SD ^1^, year)	4.7 ± 1.6
0–1 year (number, n)	13
1–3 year	20
>3 year	23
Age of onset (mean ± SD, year)	0.59 ± 1.22
>1 month (number, n)	16
1 month–1 year	20
>1 year	20
Age of initiation of sirolimus treatment (mean ± SD, year)	1.18 ± 1.44
Duration of sirolimus administration (mean ± SD, month)	18.2 ± 13.8
Previous occurrence of KMP (number, n)	
Yes	28
No	28
Platelets, hemoglobin and coagulation before catcj-up vaccination	
PLT ^2^ count (mean ± SD, 10^9^/L)	334.7 ± 88.2
Hb ^3^ concentration (mean ± SD, g/L)	121.1 ± 8.5
APTT ^4^ (mean ± SD, s)	38.9 ± 4.2
PT ^5^ (mean ± SD, s)	12.6 ± 0.5
fibrinogen (mean ± SD, g/L)	2.54 ± 0.71
D-dimer (mean ± SD, mg/L)	0.45 ± 0.31

^1^ SD, Standard deviation; ^2^ PLT, blood platelet count; ^3^ Hb, hemoglobin; ^4^ APTT, activated partial thromboplastin time; ^5^ PT, prothrombin time.

**Table 2:** Clinically observed adverse reaction following vaccination.

	Occurrences (n, %)
Mild Adverse Reaction	
Pain at the injection site	15 (1.9%)
Redness at the injection site	75 (9.6%)
Swelling at the injection site	39 (5%)
fever	8 (1%)
Severe Adverse Reaction	
Severe allergy	0 (0%)
Shock	0 (0%)
Lesion enlargement	0 (0%)
Pain at the site of the lesion	0 (0%)

Note: Pain at the injection site, crying or spontaneous pain when the limb is moved, redness at the injection site, redness and swelling measuring > 20 mm in diameter, fever, temperature > 38 °C.

**Table 3:** The number of children immunoprotected by antibody levels at various times following DTaP vaccination.

Type of Vaccine	DTaP Routine Vaccination Group (n, %)		DTaP Catch-Up Vaccination Group (n, %)	
	Post-Vaccination 0–3 Months (n = 10)	Post-Vaccination Above 6 Months (n = 8)	Post-Vaccination 0–3 Months (n = 24)	Post-Vaccination Above 6 Months (n = 18)
Pertussis	10 (100%)	8 (100%)	24 (100%)	18 (100%)
Diphtheria	9 (90%)	7 (87.5%)	23 (95.8%)	16 (88.9%)
Tetanus	9 (90%)	1 (12.5%)	24 (100%)	2 (11.1%)

Note: A pertussis IgG antibody concentration of >1 IU/mL is considered to provide long-term protection. Diphtheria IgG antibody: A concentration of >0.1 IU/mL is immunoprotective. Tetanus IgG antibody: A concentration of >1.0 IU/mL is immunoprotective.

## Data Availability

The original contributions presented in the study are included in the article/Supplementary Material. Further inquiries can be directed to the corresponding author.

## Supplementary Materials

The following supporting information can be downloaded at: https://www.mdpi.com/article/10.3390/vaccines13090903/s1, Table S1: Vaccinations required by the National Immunisation Programme (NIP) vaccine schedule for children; Table S2: Assessment of immune function in children with KHE before vaccination; Table S3: Changes in platelets, haemoglobin and coagulation in children with KHE before and after vaccination; Table S4: Hepatitis B virus surface antibody (HBs-Ab) concentration levels in hepatitis B vaccine routine and catch-up vaccination groups; Table S5: Concentration levels of IgG antibodies to pertussis, diphtheria, tetanus in the routine and catch-up vaccination groups of DTaP vaccine; Table S6: Multivariate linear regression for antibody titers at 0-3 and above 6 months post-vaccination; Table S7: Cox proportional-hazards and Kaplan–Meier survival analyses for loss of protective immunity; Table S8: Levels of antibody concentration corresponding to different doses of MMR and the number of people who reached protective antibody concentration.

## Abbreviations

The following abbreviations are used in this manuscript:

Abbreviation	Full terms
APTT	Activated Partial Thromboplastin Time
bOPV	bivalent Oral Polio Vaccine
DTaP	Diphtheria and Tetanus Toxoid with Acellular Pertussis Vaccine
DTP	Diphtheria Tetanus Pertussis Vaccine
FIB	Fibrinogen
HBs-Ab	Hepatitis B Surface Antibody
IgG	Immunoglobulin G
JE	Japanese Encephalitis
KHE	Kaposiform Hemangioendothelioma
KMP	Kasabach–Merritt Phenomenon
MMR	Measles, Mumps, and Rubella Vaccine
MRI	Nuclear Magnetic Resonance Imaging
mTOR	Mammalian target of Rapamycin
PT	Prothrombin Time
VPI	Vaccine-Preventable Infections
