# Supplementary material for: Safety and Immunogenicity of Vaccines in Children with Kaposiform Hemangioendothelioma Receiving Sirolimus: A Prospective Study
Source: Vaccines (Basel). 2025 Aug 26;13(9):903. doi: 10.3390/vaccines13090903 (PMC12474110; doi:10.3390/vaccines13090903)
Supplement: Supplementary file 1 [file vaccines-13-00903-s001.zip › vaccines-3738549-supplementary.pdf]

Supplementary Table S1 Vaccinations required by the National Immunisation Programme (NIP) vaccine schedule for children

Type of vaccine	Age-eligible individuals for vaccination (n)	Number of catch-up vaccinations (n)		Unvaccinated (n)	Vaccine deficiency rate[7] (%)
		Completing the required number of doses	Partially completed doses		
hepatitis B virus	17	36	3	0	69.6%
DTaP[1]	16	34	4	2	71.4%
MMR[2]	5	39	6	6	91%
BCG[3]	43	9	0	4	23.2%
bOPV[4]	17	33	5	1	69.6%
MPSV[5]	8	41	4	3	85.7%
JE[6]	3	32	14	7	94.6%

[1] DTaP, Diphtheria, Tetanus, and acellular Pertussis, [2] MMR, Measles, Mumps, and Rubella, [3] BCG, Bacillus Calmette–Guérin vaccine, [4] bOPV, bivalent Oral polio vaccine, [5] MPSV, Meningococcal polysaccharide, [6] JE, Japanese encephalitis [7] Vaccine deficiency rate, the absence rate is the ratio of the number of people who were not vaccinated according to the prescribed age group to the total number of people vaccinated before the vaccine was replenished. Vaccine deficiency rate = (number of people receiving catch-up vaccinations + number of people not vaccinated) / total number of people.

Supplementary Table S2 Assessment of immune function in children with KHE before vaccination

	Children with KHE treated with sirolimus (n = 56)	Reference
CD series cells		
CD8+ (%)	22.0 ± 6.5	24–34%
CD4+ (%)	45.3 ± 8.2	29–36%
CD19+ (%)	17.9 ± 4.3	14–21%
CD4+/CD8+	2.26 ± 0.95	1.5-2.0
immunoglobulin		
IgM (g/L)	1.31 ± 0.82	0.6-2.12
IgG (g/L)	5.76 ± 3.50	3.7-11.46
C3 (g/L)	1.25 ± 0.26	0.67-1.76

Supplementary Table S3 Changes in platelets, haemoglobin and coagulation in children with KHE before and after vaccination

	Pre-vaccination (n = 56)	Post-vaccination 0-3months (n = 56)	Post-vaccination above 6 months (n = 32)
PLT count (mean ± SD[1], 10^9/L)	334.7 ± 88.2	330.1 ± 77.1	343.7 ± 71.3
Hb concentration (mean ± SD[1], g/L)	121.1 ± 8.5	121.4 ± 6.6	123.7 ± 7.3
APTT (mean ± SD[1], s)	38.9 ± 4.2	38.3 ± 3.8	37.8 ± 3.1
PT (mean ± SD[1], s)	12.68 ± 0.49	12.51 ± 0.49	12.58 ± 0.51
Fibrinogen(mean ± SD1, g/L)	2.54 ± 0.71	2.76 ± 0.67	2.83 ± 0.62
D-dimer (mean ± SD[1], mg/L)	0.45 ± 0.31	0.42 ± 0.35	0.37 ± 0.21
Occurrence of KMP (n, number)	15	0	0

[1] SD, Standard deviation

Normal reference values: PLT count: 150–450×10^9/L, Hb concentration: 110–140 g/L,

APTT: 28–40 s, PT: 10–14 s, Fibrinogen (FIB): 2.0–4.0 g/L, D-dimer: <0.5 mg/L

Supplementary Table S4 Hepatitis B virus surface antibody (HBs-Ab) concentration levels in hepatitis B vaccine routine and catch-up vaccination groups

	Routine Vaccination Group[1]	Catch-up Vaccination Group[2]	t value	p value
General Information				
Numbers (n)	12	24	\	\
Age (year)	2.8 ± 1.3	1.9 ± 1.3	1.727	0.093
HBs-Antibody level (mIU/mL)				
Pre-vaccination	\	6.38 ± 4.88	6.78	< 0.01
Post-vaccination	440.61 ± 309.31	470.17 ± 335.06	-0.256	0.8
Number of cases reached protective antibody levels after vaccination[3] (n, %)	12 (100%)	24 (100%)	\	\

[1] Normal vaccination group, children who have completed 3 doses of hepatitis B vaccination prior to sirolimus administration, [2] Catch-up Vaccination Group, children who failed to complete 3 doses of hepatitis B vaccination prior to sirolimus administration but completed 3 doses of hepatitis B vaccination during sirolimus administration, [3] Concentration of Protective Antibody Levels, it is generally believed that HBs-Ab concentrations >10 mIU/mL are required for effective immunoprotection

Supplementary Table S5 Concentration levels of IgG antibodies to pertussis, diphtheria, tetanus in the routine and catch-up vaccination groups of DTaP vaccine

	DTaP routine vaccination group[1]	DTaP catch-up vaccination group[2]	t value	p value
General Information				
Numbers (n)	10	24	\	\
Age (year)	2.4 ± 1.6	2.0 ± 1.4	0.625	0.536
**Pertussis IgG antibody concentration level (IU/mL, number)**				
Pre-vaccination	-	1.60 ± 0.88 (24)	7.312	< 0.01

	DTaP routine vaccination group[1]	DTaP catch-up vaccination group[2]	t value	p value
Post-vaccination 0-3 months	33.54 ± 24.57 (10)	25.67 ± 16.14 (24)	1.106	0.277
Post-vaccination above 6 months	2.99 ± 1.26 (8)	4.32 ± 2.18 (20)	-1.608	0.12
**Diphtheria IgG antibody concentration level (IU/mL, number)**				
Pre-vaccination	-	0.018 ± 0.016 (24)	7.482	< 0.01
Post-vaccination 0-3 months	1.17 ± 0.68 (10)	1.43 ± 0.93 (24)	-0.797	0.431
Post-vaccination above 6 months	0.30 ± 0.21 (8)	0.38 ± 0.23 (18)	-0.830	0.415
**Tetanus IgG antibody concentration level (IU/mL, number)**				
Pre-vaccination	-	0.17 ± 0.24 (24)	8.669	< 0.01
Post-vaccination 0-3 months	1.99 ± 1.43 (10)	2.74 ± 1.15 (24)	-1.310	0.20
Post-vaccination above 6 months	0.39 ± 0.47 (8)	0.53 ± 0.44 (18)	-0.732	0.471

[1] DTaP routine vaccination group, children who have completed the required number of doses of DTaP before sirolimus administration and within the appropriate age range,
[2] DTaP catch-up vaccination group, children who did not complete DTaP vaccination before sirolimus administration but completed the required dose during sirolimus administration

Supplementary Table S6 Multivariate linear regression for antibody titers at 0-3 and above 6 months post-vaccination

Antibody	Time-point (month)	Age β[1] (95% CI)	p value	Sirolimus duration β[1] (95% CI)	p value
Pertussis IgG	0-3	–0.08 (–0.38, 0.22)	0.58	–0.26 (–0.51, –0.01)	0.045
Pertussis IgG	above 6	–0.11 (–0.40, 0.19)	0.47	–0.33 (–0.61, –0.05)	0.022
Diphtheria IgG	0-3	–0.02 (–0.19, 0.15)	0.84	–0.18 (–0.39, 0.04)	0.107
Diphtheria IgG	above 6	–0.04 (–0.25, 0.18)	0.72	–0.22 (–0.46, 0.03)	0.083
Tetanus IgG	0-3	–0.07 (–0.30, 0.17)	0.56	–0.30 (–0.57, –0.03)	0.034
Tetanus IgG	above 6	–0.10 (–0.36, 0.16)	0.45	–0.37 (–0.68, –0.06)	0.019

[1]β = regression coefficient which represent the change in log_{10} transformed antibody concentration (IU/mL) per 1-year increase in age or sirolimus duration.

Supplementary Table S7 Cox proportional-hazards and Kaplan–Meier survival analyses for loss of protective immunity

Antibody	HR for sirolimus duration (per year) (95% CI)	p value	Median protection time (months)	95% CI
Pertussis IgG	1.28 (1.00–1.64)	0.049	3.2	2.4–4.0
Diphtheria IgG	1.21 (0.91–1.60)	0.181	4.5	3.4–5.6
Tetanus IgG	1.40 (1.01–1.93)	0.042	5.1	3.7–6.5

Supplementary Table S8 Levels of antibody concentration corresponding to different doses of MMR and the number of people who reached protective antibody concentration

	1 dose of inoculation (n, number)			2 doses of inoculation（n, number）		
	Measles IgG antibody	Rubella IgG antibody	Mumps IgG antibody	Measles IgG antibody	Rubella IgG antibody	Mumps IgG antibody
Antibody concentration（IU/mL）						
Pre-vaccination	11.4 ± 7.4 (n = 6)	1.37 ± 0.33 （n = 6）	20.9 ± 18.1 （n = 6）	12.5 ± 14.2 (n = 30)	2.20 ± 2.36 (n = 30)	24.9 ± 17.0 (n = 30)
Post-vaccination 0-3 months	1359.9 ± 576.3 (n = 6)	58.12 ± 17.34 (n = 6)	1566.1 ± 700.0 (n = 6)	2756.2 ± 2116.2 (n = 30)	86.95 ± 49.68 (n = 30)	3300.9 ± 2754.9 (n = 30)
Post-vaccination above 6 months	1094.8 ± 298.9 (n = 5)	36.63 ± 19.28 (n = 5)	352.9 ± 281.6 (n = 5)	1928.0 ± 1372.0 (n = 24)	49.84 ± 29.81 (n = 24)	1115.4 ± 79.15 (n = 24)
Number of people with antibody levels up to the protective antibody concentration（n, %）						
Post-vaccination 0-3 months	6（100%）	6（100%）	6（100%）	30（100%）	30（100%）	30（100%）
Post-vaccination above 6 months	5（100%）	5（100%）	4（80%）	22（91.7%）	22（91.7%）	30（100%）
